# The Majority of the Pre-Antiretroviral Population Who Were Lost to Follow-Up Stopped Their Care in Freetown, Sierra Leone: A 12-Month Prospective Cohort Study Starting with HIV Diagnosis

**DOI:** 10.1371/journal.pone.0149584

**Published:** 2016-02-22

**Authors:** J. Daniel Kelly, Gabriel Warren Schlough, Sulaiman Conteh, M. Bailor Barrie, Brima Kargbo, Thomas P. Giordano

**Affiliations:** 1 Department of Medicine, Baylor College of Medicine, Houston, Texas, United States of America; 2 Wellbody Alliance, Koidu Town, Sierra Leone; 3 National HIV/AIDS Secretariat, Freetown, Sierra Leone; 4 College of Medicine and Allied Health Sciences of University of Sierra Leone, Freetown, Sierra Leone; University of Malaya, MALAYSIA

## Abstract

**Background:**

The heterogeneity of the pre-antiretroviral (pre-ART) population calls for more granular depictions of the cascade of HIV care.

**Methods:**

We studied a prospective cohort of persons newly diagnosed with HIV infection from a single center in Freetown, Sierra Leone, over a 12-month period and then traced those persons who were lost to follow-up (LTFU) during pre-ART care (before ART initiation). ART eligibility was based on a CD4 cell count result of ≤ 350 mm/cells and/or WHO clinical stage 3 or 4. Persons who attended an appointment in the final three months were considered to be retained in care. Adherence to ART was measured using pharmacy refill dates. “Effective HIV care” was defined as completion of the cascade of care at 12-months regardless of whether patients are on ART. Tracing outcomes were obtained for those who were LTFU during pre-ART care.

**Results:**

408 persons newly diagnosed with HIV infection were screened, 338 were enrolled, and 255 persons were staged for ART. ART-ineligible persons had higher retention rates than ART-eligible persons (59.6% vs 41.8%, p = 0.03). 77 (22.8%) of 338 persons received effective HIV care. Most attrition (61.9%) occurred with persons during pre-ART care. 123 of 138 persons (89.1%) who were LTFU prior to ART initiation were found, and 91 of those 123 (74.0%) were alive. Of the 74 persons who were alive and described their engagement in care, 40 (54.1%) stopped care. Nearly half (42.5%) of those 40 stopped after assessment of ART-eligibility but before ART initiation. The main limitation of this study was the lack of tracing outcomes for those lost during ART care.

**Conclusions:**

The majority of the pre-ART LTFU population stopped their care, particularly after ART-eligibility but before ART initiation. Interventions to hasten ART initiation and retain this at-risk group may have significant downstream impact on effective HIV care.

## Introduction

A decade ago, the steps of HIV care–from testing to treatment–became a visionary roadmap for controlling the epidemic as well as reducing morbidity and mortality.[1] After HIV diagnosis, patients needed to be linked to care, retained over time, initiated on antiretroviral therapy (ART) and adherent to ART in order to realize the full benefits of ART. This process later became widely known as the cascade of care.[2] The importance of the cascade of care grew as treatment was increasingly recognized as a prevention tool.[3] Yet, even after ART was made accessible around the world, a minority of HIV-infected persons succeeded in completing the cascade of care and achieving viral suppression.[4]

Barriers have been described at every step in HIV care and prevented effective use of ART.[5] In sub-Saharan Africa, the push to roll-out ART and strengthen related health systems led to a necessary focus on HIV-infected persons who successfully initiated ART. As more studies mapped the cascade of care before ART initiation, retention rates of HIV-infected persons who received pre-ART care were lower than those in persons who had initiated ART.[6] Furthermore, the higher viral loads found in HIV-infected persons who had not yet initiated ART raised public health concerns about increased transmission risks and emphasized the need for interventions for the pre-ART population.[7]

The majority of studies describing pre-ART care are retrospective in design.[6] Given the relatively weak health system for those receiving pre-ART care in sub-Saharan Africa, retrospective studies have methodological limitations, such as poor documentation of appointment attendance and CD4 cell count results. Prospective studies, however, present a separate set of challenges. Besides the inherent difficulty of following persons who are at high risk of loss to clinical follow-up, pre-ART care consists of multiple observational periods (i.e., diagnosis to initial clinical visit, ART-ineligibility to retention in pre-ART care, and ART-eligibility to ART initiation) in comparison to the consistent observational period during ART care. The complexity of pre-ART care has resulted in most studies describing some but not all of these observational periods, or other studies describing all observational periods through different cohorts.[6–8]

In order to better understand what happens to persons lost to care, some studies employ tracers to find persons out of care and ascertain their vital status. Few studies have traced persons who were lost to follow-up (LTFU) during pre-ART care and that population was limited to those who were LTFU after being assessed for ART-eligibility.[9, 10] To our knowledge, no study has followed newly diagnosed persons through the cascade of care and systematically traced persons who were LTFU during each step of pre-ART care (prior to ART-staging, after staged as ART-eligible, after staged as ART-ineligible). The goal of our study was to prospectively follow a cohort of newly diagnosed persons with HIV infection through the cascade of care for 12-months and trace the outcomes of those who were LTFU during pre-ART care.

## Methods

### Ethics statement

Written informed consent was obtained from all patients enrolled in the study. The consent form was read to illiterate participants, and the participants made a thumbprint ink mark to indicate their agreement to enroll in the study. After enrollment, a survey was administered at the time of new diagnosis. Participants were compensated USD 1, roughly the average transportation cost of a round-trip to the HIV clinic, at the time of survey completion. This study and consent procedure was approved by the Institutional Review Board of Baylor College of Medicine and Sierra Leone Ethics and Scientific Committee.

### Participants

We sequentially recruited a prospective cohort of persons newly diagnosed with HIV infection from July 2011 to March 2012 at Connaught Hospital in Freetown, Sierra Leone. Freetown is the capital of Sierra Leone, and people in Freetown typically speak either English or Krio. Connaught Hospital is a tertiary-care public sector health facility with the largest HIV clinic in Sierra Leone. More than 3,000 patients attend the HIV clinic. It has co-localized services, including Voluntary Confidential Counseling and Testing (VCCT), CD4 cell count testing capacity, pharmacy for dispensing ART and prophylaxis for opportunistic infections, and a consulting room for health providers.

Persons presenting to the HIV clinic at Connaught Hospital needed to meet the following eligibility criteria to be enrolled in our study: 1) a new diagnosis of HIV infection, defined as being diagnosed with HIV less than 3 months before a current, confirmatory diagnosis AND not yet having completed an initial HIV clinic appointment, 2) ability to speak English or Krio, 3) ability to provide informed consent (or have a representative able to do so), 4) age greater than or equal to 18 years, and 5) willingness and having cognitive ability to complete the survey, in the discretion of the research team. We excluded people with prior exposure to ART or the stated intention to receive HIV care at another facility. After screening, some patients were subsequently found to have the following unreported events and were also excluded: 1) HIV diagnosis greater than 90 days before screening, 2) prior exposure to ART, and 3) a prior HIV clinical appointment. Additional participants transferred their HIV care to another facility during the study and were excluded because of the challenges tracing their retention in care at the new facility. Participants were excluded if their personal information was lost from the record management system of the clinic. Those participants who died during the study were excluded from the analysis conducted on the steps towards effective HIV care.

### Data collection and outcomes

Participants were followed during as many as three observational periods: 1) from HIV diagnosis to ART staging, 2) after ART ineligibility (if applicable), and 3) after ART eligibility. Data on HIV testing, ART eligibility and initiation, appointments, and adherence to ART were collected as described below. Additional descriptive data on participants was collected from the patient chart such as ‘disclosed HIV status.’

ART eligibility was based on a CD4 cell count result of ≤ 350 mm/cells and/or WHO clinical stage 3 or 4. ART initiation was defined as being ART eligible and having completed all of the pre-treatment steps (i.e., exclusion of co-morbid acute illnesses, expressed willingness to take ART, completion of adherence counseling) necessary for the HIV health provider to prescribe ART. ART ineligibility was based on a CD4 cell count result of ≥ 350 mm/cells and/or WHO clinical stage 1 or 2. ART ineligible persons were expected to attend monthly appointments to refill co-trimoxazole and for health checks. Co-trimoxazole (also known as Bactrim or Septra) was given as prophylaxis for opportunistic infections and to reduce morbidity in a population with a falling CD4 cell count and uncontrolled viremia. Patients who arrived to the ART and co-trimoxazole dispensary appearing sick were referred for clinical visits. Health providers re-assessed ART-ineligible persons and their need for ART by CD4 cell count testing at 6-month intervals when appearing healthy and/or WHO clinical staging when appearing sick.

Pre-ART care was defined as the observational period prior to ART initiation while ART care was defined as the observational period after ART initiation. Attendance at an appointment was defined as an appointment kept out of an appointment scheduled. Participants were followed for 12 months, and if persons did not attend an appointment to receive ART and/or prophylaxis for opportunistic infections in the final three months, or more than 90 days elapsed after a missed appointment, they were considered lost to follow-up (LTFU).[11] Retention in care was defined as the percentage of participants who attended HIV clinic to receive ART and/or prophylaxis for opportunistic infections during the final three months of the 12-month follow-up period.[12] The definition of LTFU and retention in care were the same for pre-ART and ART population.[6]

Adherence to ART was measured using pharmacy refill data. Adherence was defined as the (number of refills obtained over time)/(number of months of follow-up after removing months in which the person was LTFU). Using this definition, we classified adherence as less than 70%, 70% to less than 80%, 80% to less than 90%, 90% to less than 95%, and 95% or greater.[13] Our study population was considered adherent to ART if they took ART ≥ 90%.

**Fig 1:**
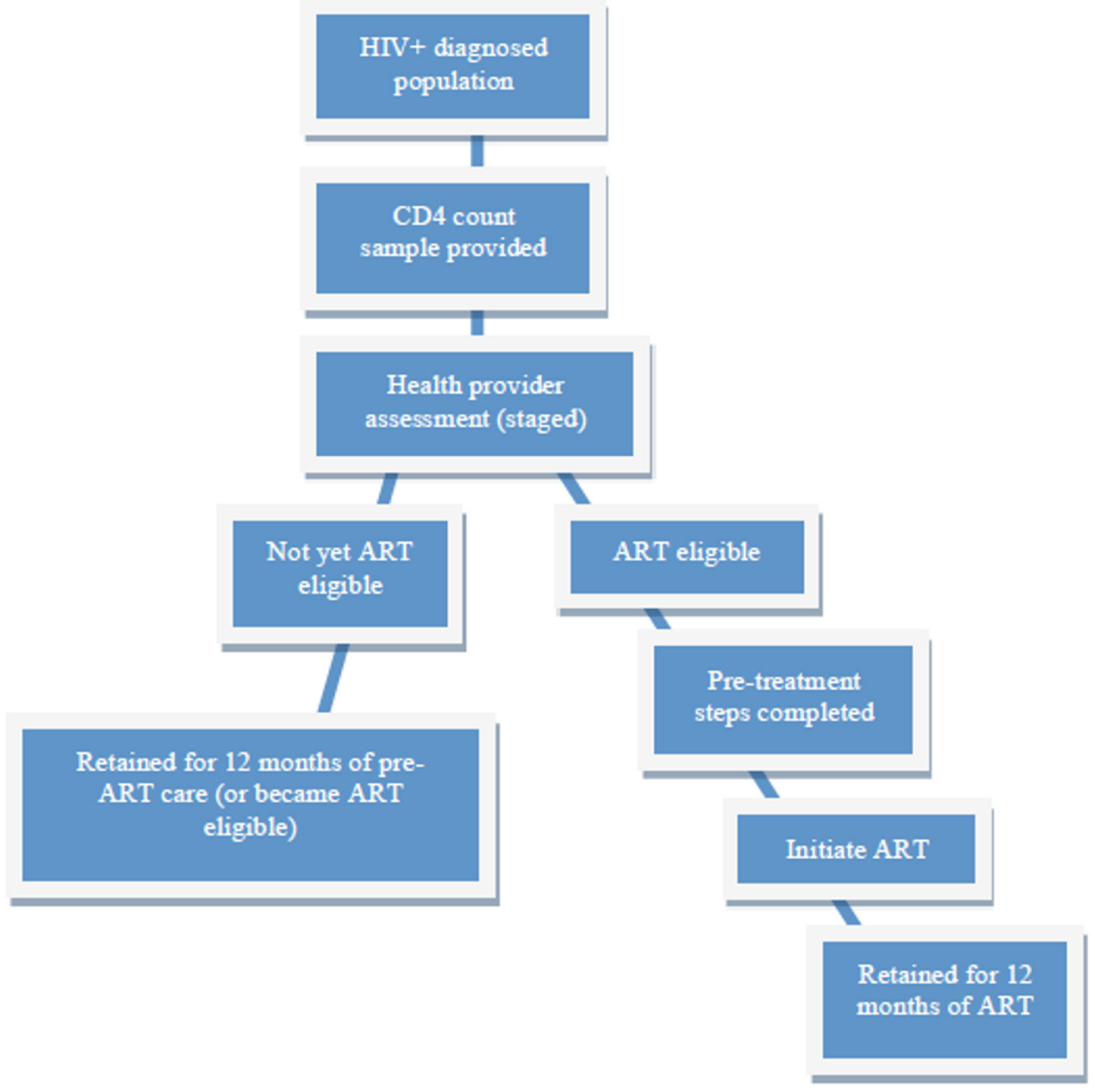
Cascade of HIV care from testing to retention in care for ART-eligible and ART-ineligible persons at 12 months.

“Effective HIV care” was defined as newly diagnosed persons with HIV infection who received a CD4 cell count result, were staged for ART within 90 days, were retained in pre-ART or ART care over 12 months based on ART-eligibility and initiation of ART within 90 days, and demonstrated ≥ 90% adherence to ART if ART was initiated (Fig 1).

We obtained additional data on the outcomes of HIV-infected persons who were LTFU during pre-ART care (defined as the observational period from HIV diagnosis to ART initiation), the National AIDS Control Program had a tracing program, and tracing was conducted as per standard of care. A local study staff member who was approved by the National AIDS Control Program accompanied the tracer during his activities to ensure quality control. Vital status was ascertained as alive or dead for all people who were successfully traced. Additional outcomes relating to engagement in care (stopped care or self-transferred care) were obtained for those who were alive. Tracing data was not collected for participants who initiated ART and then were LTFU.

### Data analysis

We calculated age, gender, educational attainment, income, disclosure of cell phone number, distance from clinic, ‘ever’ usage of tobacco and alcohol, and disclosure of HIV status as descriptive statistics for the cohort. Bivariable and multivariable regression analyses were conducted comparing those who were LTFU and retained in care using STATA/IC 13.1 (College Station, TX). We considered two-sided P values < 0.05 statistically significant, and < 0.10 indicative of a trend.

## Results

408 persons newly diagnosed with HIV infection were screened, and 338 were enrolled. Reasons for exclusion (n = 70) were the following: having prior exposure to ART (n = 19), being diagnosed with HIV infection >90 days (n = 18), intending to receive HIV care at another facility (n = 11), transferring to another facility (n = 7), medical record was lost within the patient tracking system (n = 5), having died (n = 5), and attending a prior HIV clinical visit (n = 3). Based on the available data among the 338 participants, the average age was 32, and nearly three-quarters (71.4%) were women. Over one-third (36.7%) had six-years of primary school education or less. The overwhelming majority (82.2%) made less than 1 USD per day. Few participants ever used tobacco (8.9%) or alcohol (4.8%) (Table 1).

**Table 1:** Demographic data of study population.

	338 newly diagnosed persons with HIV infection who were enrolled % (n/N)
Age^^^	32 (276)
Women	71.4 (225/315)
≤ Primary school	36.7 (114/311)
Income (≤ USD 1 per day)^^^	82.2 (203/247)
Disclosed cell number	65.1 (203/312)
Living outside Freetown	46.0 (138/300)
Ever used tobacco	8.9 (28/313)
Ever used alcohol	4.8 (15/313)
Disclosed HIV status^#^	56.3 (126/224)

^^^Many patients did not know their age, and others did not want to disclose their income

^#^This variable was obtained from patient charts while all other variables in the table were collected from the survey.

### HIV diagnosis to effective HIV care

In our cohort of 338 newly diagnosed persons with HIV infection, 326 (96%) participants provided blood for their CD4 cell count test, and a result was obtained. The median CD4 cell count of these 326 persons was 256 cells/mm3 (IQR 2–1408). It was unclear how many of these participants learned of their CD4 cell count results, but 255 (75.4%) persons attended an initial HIV clinical appointment and were assessed for ART-eligibility. The median CD4 cell count of the 255 persons who were assessed for ART eligibility was 250 cells/mm3 (SD+/-229) while the median CD4 cell count of the 71 persons who were not assessed for ART eligibility was 250 cells/mm3 (SD+/-292).

Of the 255 persons who were staged, 47 (18.4%) persons were ART-ineligible. 8 (17.0%) of 47 ART-ineligible persons became ART-eligible during the follow-up period. In those 8 persons, ART-eligibility was determined by clinical presentation in 7 cases and by CD4 cell count in 1 case. That one person was the only person of the 47 initially ART-ineligible persons who had a repeat CD4 cell count. 27 (57.4%) of the 47 persons were retained in care at 12-month follow-up.

208 (81.6%) of 255 ART-staged persons were ART-eligible. Of those 208 ART-eligible persons, 172 (82.7%) initiated ART and 87 (41.8%) were retained in care. Based on the 255 HIV-infected persons staged for ART, ART-ineligible persons had higher retention rates than ART-eligible persons (57.4% vs 41.8%, p<0.01). In total, 114 of 338 people (33.7%) were retained in care.

**Fig 2:**
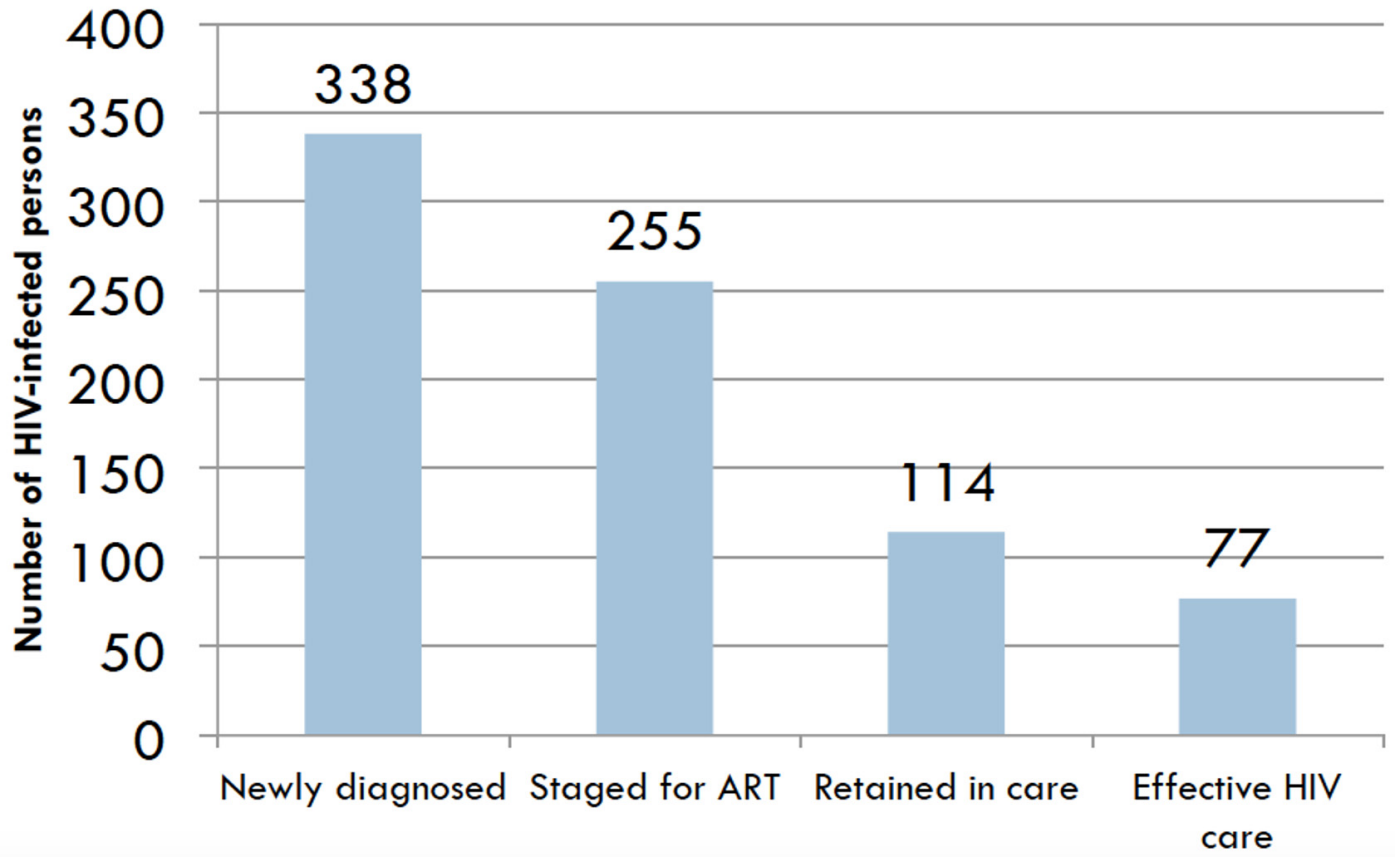
Cascade of care for newly diagnosed persons to receive effective HIV care.

Of the 87 persons who were retained in ART care, 50 (57.5%) persons were adherent to ART. These 50 persons succeeded in optimizing the cascade of effective HIV care from the cohort of persons who were ART-eligible while the 27 persons who were retained in pre-ART care at 12 months also succeeded in optimizing the cascade of effective HIV care. Together, 77 (22.8%) of 338 persons who were newly diagnosed with HIV infection received effective HIV care (Fig 2).

### Lost to follow-up through the cascade of care

During the cascade of care, attrition rates depended on the observational period. 83 (24.6%) of 338 persons were lost after HIV diagnosis but before ART staging. The median CD4 cell count for this LTFU group was 284 cells/mm3 (IQR 2–1408). 20 (5.9% of all patients and 42.6% of the patients who were ineligible) were lost after ART-ineligibility but before effective HIV care; 36 (10.6% of all patients and 17.3% of the patients who were eligible) were lost after ART-eligibility but before ART initiation; and 85 (25.1% of all patients and 49.4% of the patients who initiated ART) were lost after ART initiation but before effective HIV care. In total, 224 (66.3%) of 338 persons were LTFU, and 139 (62.3%) of 223 persons were LTFU during pre-ART care (83 before staging, 20 after being found ineligible for ART, and 36 after being found eligible but before starting ART).

In unadjusted analyses, female participants had a lower risk of being LTFU than male participants (Table 2). After a multivariable adjustment, the association between female participants and LTFU held (Adjusted Odds Ratio [AOR] = 0.46; 95% CI, 0.23–0.91). Participants living outside Freetown had a significant association with the risk of being LTFU as compared to participants living inside Freetown (AOR = 0.21; 95% CI, 0.08–0.57). We also found that participants who disclosed their HIV status had a lower risk of being LTFU than participants who did not disclose their HIV status (AOR = 0.26; 95% CI, 0.09–0.74).

**Table 2:** Factors Associated With Loss to Follow-up From HIV Clinic.

Characteristics		Unadjusted OR (95% CI)	p-value	Adjusted OR (95% CI)	p-value
Age		0.99 (0.97–1.01)	0.32	0.99 (0.96–1.01)	0.43
Gender					
	Male	Ref	0.05	Ref	0.03
	Female	0.58 (0.33–1.00)		0.46 (0.23–0.91)	
Education					
	≤ Primary school	Ref	0.20	Ref	0.12
	> Primary school	1.42 (0.83–2.42)		1.67 (0.87–3.19)	
Income					
	≤ USD 1 per day	Ref	0.28	Ref	0.80
	> USD 1 per day	0.71 (0.38–1.32)		0.90 (0.41–1.98)	
Disclosed cell number					
	No	Ref	0.80	Ref	0.14
	Yes	1.07 (0.65–1.75)		2.11 (0.78–5.69)	
Distance from clinic					
	Inside Freetown	Ref	0.08	Ref	<0.01
	Outside Freetown	0.65 (0.40–1.05)		0.21 (0.08–0.57)	
Ever use tobacco					
	No	Ref	0.31	Ref	0.07
	Yes	0.67 (0.31–1.45)		0.21 (0.04–1.16)	
Ever used alcohol					
	No	Ref	0.25	Ref	0.54
	Yes	0.54 (0.19–1.54)		2.23 (0.17–28.75)	
Disclosed HIV status					
	No	Ref	0.63	Ref	0.01
	Yes	0.86 (0.48–1.57)		0.26 (0.09–0.74)	

### Tracing outcomes of HIV-infected people lost to follow-up during pre-ART care

Of the 139 people who were LTFU during pre-ART care, 123 (89.1%) were found and the vital status was ascertained. 91 (74.0%) were alive and 32 (26.0%) were dead. Of the 32 persons who died, 13 (40.6%) died after HIV diagnosis but before ART staging, 6 (18.8%) died after ART-ineligibility, and 13 (40.6%) died after ART-eligibility but without initiating ART.

74 (81.3%) of 91 persons who were alive had additional outcomes. 40 (54.1%) had stopped care, and 34 (45.9%) had self-transferred care from the HIV clinic at Connaught Hospital to another facility. Of the 40 persons who stopped care, 12 (30.0%) stopped care after HIV diagnosis but before ART staging, 11 (27.5%) stopped after ART-ineligibility, and 17 (42.5%) stopped after ART-eligibility but before ART initiation. Of the 34 persons who were lost from the HIV clinic at Connaught Hospital and found to have self-transferred care, 11 (32.4%) self-transferred care after HIV diagnosis but before ART staging, 6 (17.6%) self-transferred after ART-ineligibility, and 17 (50.0%) self-transferred after ART-eligibility but before ART initiation. Within the group of persons who were ART-staged and then self-transferred care, ART-eligible persons were more likely to self-transfer care than ART-ineligible persons (50.0% vs 17.6%, p = 0.05). The tracer was unable to investigate their retention in other facilities.

## Discussion

Here, we present the first depiction of the cascade of HIV care in Sierra Leone. We found most attrition occurred during pre-ART care (61.9%); the majority of the pre-ART LTFU population stopped their care (54.1%), particularly after ART-eligibility but before ART initiation; and less than a quarter of newly diagnosed persons optimized the steps needed to receive effective HIV care (22.8%). Given this granular depiction of the cascade of care, our findings suggest HIV-infected persons who stopped pre-ART care represent a major public health threat to efforts to end the AIDS epidemic. Although the World Health Organization recently recommended ART for all HIV-infected persons,[14] the change towards universal access to ART will take time for many countries, and targeted structural and behavioral interventions will need to accompany expansion efforts, if the pre-ART LTFU population is to benefit from modern medicine.[15] Together, these interventions may demonstrate significant downstream impact on effective HIV care for the pre-ART LTFU population by preventing secondary transmission of HIV infections and improving long-term clinical outcomes.[16]

Poor retention in pre-ART care has been generally described, but attrition within different pre-ART periods of care is less well understood.[6–8] In particular, few studies follow a cohort of newly diagnosed persons with HIV infection throughout all observational periods. In our study as well as others, persons who were newly diagnosed with HIV infection and not yet staged for ART had the highest rate of attrition as compared to other pre-ART observational periods.[8, 17] After ART staging and the 12-month observational period, we found high retention rates (57.4%) for ART-ineligible persons. Lower retention rates have been found in prior studies of ART-ineligible persons, and this discrepancy may point to an under-estimation of retained persons. Imperfect measures of retention such as repeat CD4 cell counts and attendance at clinic appointments can misclassify healthy persons arriving solely to receive refills for opportunistic infection prophylaxis. In contrast to ART-ineligible persons, we noted significant attrition of ART-eligible persons during the 12-month observational period, resulting in a relatively lower retention rate of 41.8%. Over two-thirds (70.2%) of ART-eligible persons were lost after ART initiation, and this ART LTFU population accounted for over one-third (38.1%) of all persons lost to follow-up. While this attrition was notable, other studies tracing ART LTFU outcomes have documented that this population was highly likely to be found alive and self-transfer care [18], suggesting the need to focus attention on pre-ART LTFU populations.

Our study traced the outcomes of persons who were lost to follow-up during pre-ART care, and we found over a quarter died (26.0%) and over half stopped care (54.1%). Since our study was conducted, Namusboya et al. reported findings supporting the predisposition for the pre-ART LTFU population to stop care, though this study did not include people who were LTFU before being assessed for ART-eligibility.[10] Our study, however, deepened knowledge of tracing outcomes in the pre-ART population because it draws attention to the sub-population most likely to stop care: ART-eligible persons not yet initiated on ART. Although our sample size is small, our results suggest that interventions to initiate more people on ART will move persons down the cascade of HIV care. Furthermore, even if this group became LTFU after ART initiation, tracing studies suggest they would be more likely to self-transfer care and thus have a greater chance of effective HIV care.

Data from this study were obtained from a single center located at the adult hospital in Freetown. The maternal and child hospitals in Freetown, for example, have independent HIV clinics and were not included in this study. Extrapolation of findings beyond the HIV clinic of Connaught Hospital should be approached with caution. We recognize tracing data can counterbalance the limitations of retention rates from a single center. The tracing part of the study, however, did not include persons who had initiated ART, and we did not have enough data to calculate sample-weighted outcomes. Financial limitations in the setting of a pre-existing body of literature precluded additional investigation of the ART LTFU population.[8, 18] The strength of this study was the granular detail with which we prospectively followed and traced one cohort of 338 persons from diagnosis to effective HIV care with every observational period in between noted, even as LTFU participants moved in cyclic patterns when they re-engaged in care.

Our granular depiction of the cascade of care at this large HIV clinic in Freetown, Sierra Leone, underscore the importance of conceptualizing roadmaps for cascade interventions while considering both the clinic and the network of surrounding communities and clinics. A clinic-based retention intervention may be considered for HIV-infected persons who are not yet staged for ART while the ART-eligible persons who stopped care may benefit from a community-based intervention and more intensive tracing with counseling. Qualitative work is needed to understand the associations reported in this study; this work is also more broadly needed to understand the barriers to pre-ART care, inform potential interventions, and add to the dearth of literature on the topic.[19] Our evidence supports the need to move beyond a general strategy for pre-ART retention interventions.[7]

By demonstrating 22.8% of the Sierra Leone population received effective HIV care, this study provided a new lens on the cascade of care for the global HIV community. The Ebola epidemic, however, created setbacks to build on prior HIV response efforts. As Sierra Leone moves beyond Ebola, this study can serve as a baseline for the cascade of HIV care and a needs assessment for targeted interventions as HIV programs re-engage Ebola-affected communities.
